# Urgent Splenectomy after Partial Splenic Embolization in Liver-Transplanted Patient: A Case Report

**DOI:** 10.1155/2012/959635

**Published:** 2012-11-10

**Authors:** Jorge Herrador Benito, M. G. Zunzarren, T. Pozancos de Simón, L. Tortolero, R. Latorre Fragua, J. Nuño, E. Lobo

**Affiliations:** ^1^Department of General and Digestive Surgery, Ramón y Cajal Hospital, Cra. Colmenar Km 9, 1, 28034 Madrid, Spain; ^2^Liver Transplantation Unit, Department of General and Digestive Surgery, Ramón y Cajal Hospital, Cra. Colmenar Km 9, 1, 28034 Madrid, Spain

## Abstract

We describe a 51-year-old-male. Three months after liver transplantation due to hepatitis C virus (HCV) hepatopathy, an HCV relapse was detected, and partial splenic embolization (PSE) was performed prior to antiviral treatment. Eleven days after PSE emergency splenectomy was performed due to the development of a splenic abscess, which is a rare but severe complication of PSE. Between May 2002 and March 2012, 18 PSEs have been performed in transplant patients in our centre. The patient presented here is the only case of splenic abscess and the only one who has needed surgery after complications of PSE.

## 1. Introduction

Approximately 20–30% of patients who receive a liver transplant due to hepatitis c virus hepatopathy develop cirrhosis before 5 years, and up to 14% experience a serious relapse in the first year after transplantation [1–3]. Hypersplenism in these patients results in decreased platelet levels, which does not allow treatment with pegylated interferon (Peg-IFN) and ribavirin because these drugs are associated with hematological toxicity, especially thrombocytopenia in the case of Peg-IFN. Partial splenic embolization (PSE) is an effective alternative to splenectomy in these patients to correct platelet levels if antiviral treatment is necessary [4–7].

## 2. Case Report

We describe a 51-year-old male who received a liver transplant secondary to HCV liver cirrhosis. Immunosuppression received was Tacrolimus 6 mg/12 h and Methylprednisolone 20 mg. During the postoperative period the patient presented with right pleural effusion, mild renal failure, and mild preservation injury with minimal graft dysfunction compatible with preservation cholestasis.

Three months after liver transplantation a serious HCV relapse was detected by the presence of serious lobular hepatitis in a liver biopsy, with a total bilirubin of 6 mg/dL, HCV viral load of 100,000,000 UI/mL, and hyperglycemia associated with the relapse. Platelet levels were 44,600 cells/mm^3^ (see Table 1). 

**Figure 1:**
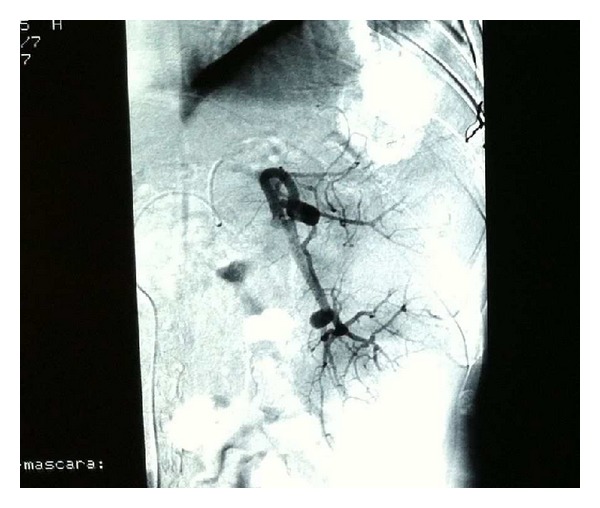
Splenic arteriography.

To correct the blood platelet levels prior to antiviral treatment, splenic embolization was performed for approximately 90% of the parenchyma, preserving only a central hilar area and another area in the upper pole (see Figure 1). The technique was distal superselective catheterization of the splenic artery through the femoral artery with the injection of particles of polyvinyl alcohol in solution with penicillin, gentamicin, and iodinated contrast. The patient was discharged 6 days after embolization, after a period of time without complications.

Three days later, the patient arrived at the emergency department with a fever of 38.5°C and diffuse abdominal pain, primarily in the left upper quadrant. An urgent CT scan showed free fluid in the pelvis and paracolic gutters, with uptake of contrast medium in the peritoneum. The spleen was enlarged with gas bubbles compatible with the embolization.

**Figure 2:**
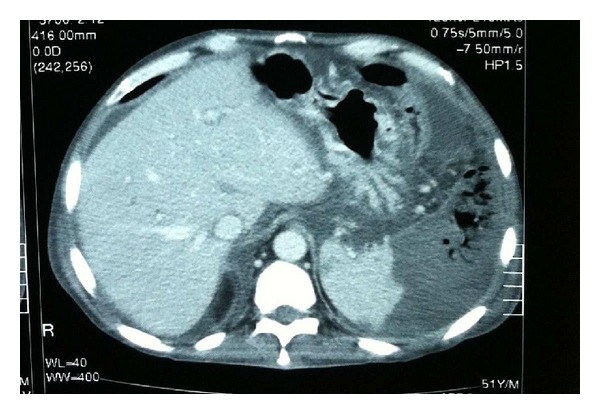
CT scan.

We decided to initiate empiric antibiotic and antimycotic treatment (meropenem, tobramycin, and fluconazole). Over the following days, the clinical course included torpidity, development of renal failure, leukocytosis above 30,000, and persisting malaise with abdominal pain and fever. At all times the patient was hemodynamically stable with no need for vasoactive drugs and without respiratory support. *Bacteroides fragilis* was isolated in blood culture. Eleven days after admission, a new CT scan showed multiple splenic collections of gas compatible with a splenic abscess secondary to the PSE. In addition, adjacent hemoperitoneum was observed, and the splenic capsule was ruptured (Figure 2). 

Emergency surgery found roughly 3.5 liters of reddish-brown peritoneal fluid. Splenectomy was performed and blood products transfused. After 3 days in the ICU, the patient required a prolonged stay and was discharged at postoperative day 40. Regarding the evolution of graft function from the time of embolization, the analytical figures were gradually normalized, reaching the following levels: AST = 43, ALT = 16, FA = 202, and GGT = 296.

## 3. Discussion

The morbidity of PSE is well known. Almost 100% of patients develop the so-called “postembolization syndrome” (fever, abdominal pain, nausea) caused by splenic infarction and the release of proinflammatory cytokines. Other “minor” complications were pleural effusion, the development of neutrophilic ascites, which is usually transient and responsive to diuretic therapy, and splenic or portal thrombosis. The most serious complications are the development of splenic abscess and distal pancreatitis. Splenic abscess formation is not a infrequent and severe complication of PSE that occurs in 5–15% of PSE. Like other complications, abscess formation is related to the volume of parenchyma that is embolized, occurring with volumes greater than 70%. The treatment of choice is urgent splenectomy; though medical treatment support, antibiotics, and occasional percutaneous drainage can be attempted in patients with major functional impairment, it often provides worse results [8–10].

Between May 2002 and March 2012, 74 PSEs have been performed in our centre, including 18 procedures in transplant patients. The patient presented here is the only case of splenic abscess, and the only one who has required surgery after complications of PSE, assuming 1.4% for total PSE and 5.6% among transplant patients.

## Figures and Tables

**Table 1:** The evolution of blood platelet levels.

	Platelets (cells/mm^3^)
Prembolization levels (70 days post-transplant)	44,600
Postembolization levels (15 days post-PSE)	318,000
Postsplenectomy (38 days after splenectomy)	466,000
